# Individualizing isotretinoin dosing in acne: comparable 24-week efficacy and better tolerability at lower daily doses

**DOI:** 10.3389/fmed.2026.1771320

**Published:** 2026-03-09

**Authors:** Jingmin Zou, Yuxing Li, Lu Wang, Ying Jiang, Fumin Fang, Kai Shao, Xiaoping Zhang, Tingting Zhu, Xiuqin Yu

**Affiliations:** Department of Dermatology, The First Affiliated Hospital of Soochow University, Suzhou, China

**Keywords:** acne vulgaris, cumulative dose, isotretinoin, low-dose therapy, relapse, systematic review and meta-analysis

## Abstract

**Background:**

The optimal isotretinoin dosing strategy for acne vulgaris remains debated, balancing efficacy against dose-dependent toxicity.

**Objective:**

To compare the efficacy, safety, and post-treatment outcomes of low-dose (≤0.5 mg/kg/day) versus conventional-dose (0.5–1.0 mg/kg/day) isotretinoin.

**Methods:**

We searched PubMed, Embase, CENTRAL, and Web of Science for randomized controlled trials (RCTs). The primary outcome was the change in Global Acne Grading System (GAGS) score at 24 weeks. Secondary outcomes included post-treatment worsening quantified as GAGS score deterioration, adverse events, and patient satisfaction. Certainty of evidence was assessed using GRADE.

**Results:**

Four trials (210 randomized; 202 analyzed) were included. At 24 weeks, the pooled mean difference favored neither regimen [MD = −1.87, 95% CI (−4.38, 0.64); *p =* 0.15; *I*^2^ = 85.67%]. Heterogeneity was predominantly attributable to one trial that enrolled more severe cases and used adjunctive antibiotics/corticosteroids; excluding it reduced heterogeneity and maintained no significant difference under a random-effects model [MD = −0.92, 95% CI (−2.62, 0.77); *p =* 0.28; *I*^2^ = 44.97%]. Post-treatment worsening did not differ overall and, in sensitivity analyses excluding the co-intervention trial, showed no statistically significant difference with no heterogeneity [MD = 0.10, 95% CI (−1.67, 1.86); *p =* 0.91; *I*^2^ = 0%]. Low-dose regimens had higher patient satisfaction [SMD = 0.99, 95% CI (0.63, 1.34); *p* < 0.001] and better tolerability. Certainty of evidence was low for efficacy and moderate for safety and satisfaction (GRADE).

**Conclusion:**

In RCTs conducted predominantly in moderate acne and in contexts minimizing co-interventions, low-dose isotretinoin provides comparable 24-week efficacy to conventional dosing while offering superior tolerability and higher patient satisfaction. Conventional dosing may yield a modestly faster early response; however, the absolute differences were small and evidence in substantially more severe acne remains limited and context-dependent.

**Systematic review registration:**

Registered in PROSPERO (ID: CRD42024536322), URL: https://www.crd.york.ac.uk/PROSPERO/view/CRD42024536322.

## 1 Introduction

Acne vulgaris is a highly prevalent chronic inflammatory skin disorder of the pilosebaceous unit that significantly affects individuals’ quality of life, particularly during adolescence (1). The condition is characterized by the presence of comedones, papules, pustules, and, in more severe cases, nodules and cysts, frequently resulting in scarring and psychological distress (2, 3). Distinct therapeutic strategies including various topical, oral and physical treatments are available in relation to acne severity. Despite the emergence of novel therapies, isotretinoin remains the gold standard for severe nodulocystic acne (4).

Since FDA approval in 1982, oral isotretinoin remains the only therapy targeting all four pathogenic mechanisms: sebum hyperproduction, abnormal follicular keratinization, Cutibacterium acnes proliferation, and inflammation (5–9). Traditional dosing guidelines, recommending 0.5–1.0 mg/kg/day to reach a cumulative dose of 120–150 mg/kg, were largely derived from early experience with severe nodulocystic acne (10–12). While effective, this conventional regimen is associated with dose-dependent mucocutaneous and laboratory adverse effects (11, 13), which can impair adherence and quality of life (14). These limitations have prompted investigation of alternative dosing strategies, particularly for moderate acne where conventional doses may represent overtreatment (15, 16). Studies suggest low-dose regimens achieve similar therapeutic outcomes with improved tolerability (17–23).

However, adoption has been hindered by conflicting data regarding remission and by non-standardized outcome measures across trials. We conducted this systematic review and meta-analysis using standardized outcomes to clarify the comparative effectiveness of low-dose versus conventional-dose isotretinoin, focusing on the GAGS change as our primary metric. Specifically, we quantify “relapse” as the change in GAGS score between the end of follow-up and the end of treatment, enabling a more objective analysis. This meta-analysis systematically compares efficacy, safety, patient satisfaction, treatment dynamics and post-treatment outcomes between dosing regimens.

## 2 Methods

### 2.1 Protocol and registration

This systematic review and meta-analysis was conducted in accordance with the Preferred Reporting Items for Systematic Reviews and Meta-Analyses (PRISMA) 2020 statement and was registered in PROSPERO (CRD42024536322).

### 2.2 Search strategy and study selection

We systematically searched PubMed, Embase, Cochrane CENTRAL, and Web of Science from inception to 17 May 2025, without language restrictions. The search strategy combined controlled vocabulary (MeSH/Emtree) and free-text terms for “isotretinoin,” “acne vulgaris,” and “dose.” Full search strings are detailed in Supplementary File S1. Two reviewers independently screened records, with a third reviewer adjudicating disagreements.

### 2.3 Eligibility criteria

Studies were selected according to the PICO framework: (1) Design: RCTs; (2) Population: patients with acne vulgaris; (3) Intervention: low-dose oral isotretinoin (≤0.5 mg/kg/day or fixed dose ≤20 mg/day); (4) Comparator: conventional-dose oral isotretinoin (0.5–1.0 mg/kg/day); (5) Outcomes: studies must report GAGS score data suitable for quantitative analysis.

### 2.4 Data extraction and quality assessment

Data were extracted independently by two reviewers. Data were extracted based on the intention-to-treat (ITT) principle whenever reported by the primary authors [e.g., Lee et al. (24)]. For studies where ITT data were not available or imputation was not performed [e.g., Kassem et al. (25)], data from the per-protocol (completer) population were utilized. Risk of bias was assessed using the Cochrane Risk of Bias 2 (RoB 2) tool at the outcome level. We assessed five domains: randomization process, deviations from intended interventions, missing outcome data, measurement of the outcome, and selection of the reported result. Visualizations were generated using the robvis tool.

### 2.5 Data synthesis and statistical analysis

To standardize post-treatment outcomes across trials reporting follow-up GAGS, relapse was quantified as “GAGS score deterioration,” defined as the absolute increase in GAGS score from the end of treatment to the end of the follow-up period. Studies reporting only dichotomous relapse [e.g., El Aziz Ragab et al. (26)] without quantitative follow-up GAGS data were excluded from this continuous synthesis. This operational definition facilitates quantitative synthesis but differs from relapse definitions used in primary trials[e.g., retreatment-based relapse in El Aziz Ragab et al. (26), and categorical deterioration to moderate-or-worse acne based on GAGS thresholds in Lee et al. (24)]. Accordingly, relapse findings in this review should be interpreted as changes in severity scores rather than treatment-requiring recurrence, which may limit external validity and comparability with prior literature. We used random-effects (REML) models to calculate mean differences (MD) for continuous outcomes and standardized mean differences (SMD) for patient satisfaction (due to scale variations). For trials reporting intermediate assessments, we additionally synthesized between-group differences in GAGS improvement (change from baseline) at weeks 12, 16, and 20 using random-effects (REML) models to describe treatment kinetics.

For studies reporting baseline and follow-up means without change scores, we imputed the SD of the change using an assumed within-participant correlation (*r* = 0.5), following the recommendations of the Cochrane Handbook (27); robustness was assessed in sensitivity analyses using *r* = 0.3 and *r* = 0.7. Pre-specified sensitivity analyses included leave-one-out and fixed-effect versus random-effects modeling. Given the small number of included studies (*n* < 10), Egger’s test was not performed as it would have insufficient statistical power. Analyses were performed using Stata 16.

Finally, the certainty of evidence for key outcomes was assessed using the Grading of Recommendations Assessment, Development and Evaluation (GRADE) approach, classifying evidence as high, moderate, low, or very low quality.

## 3 Results

### 3.1 Study selection and characteristics

**Figure 1:**
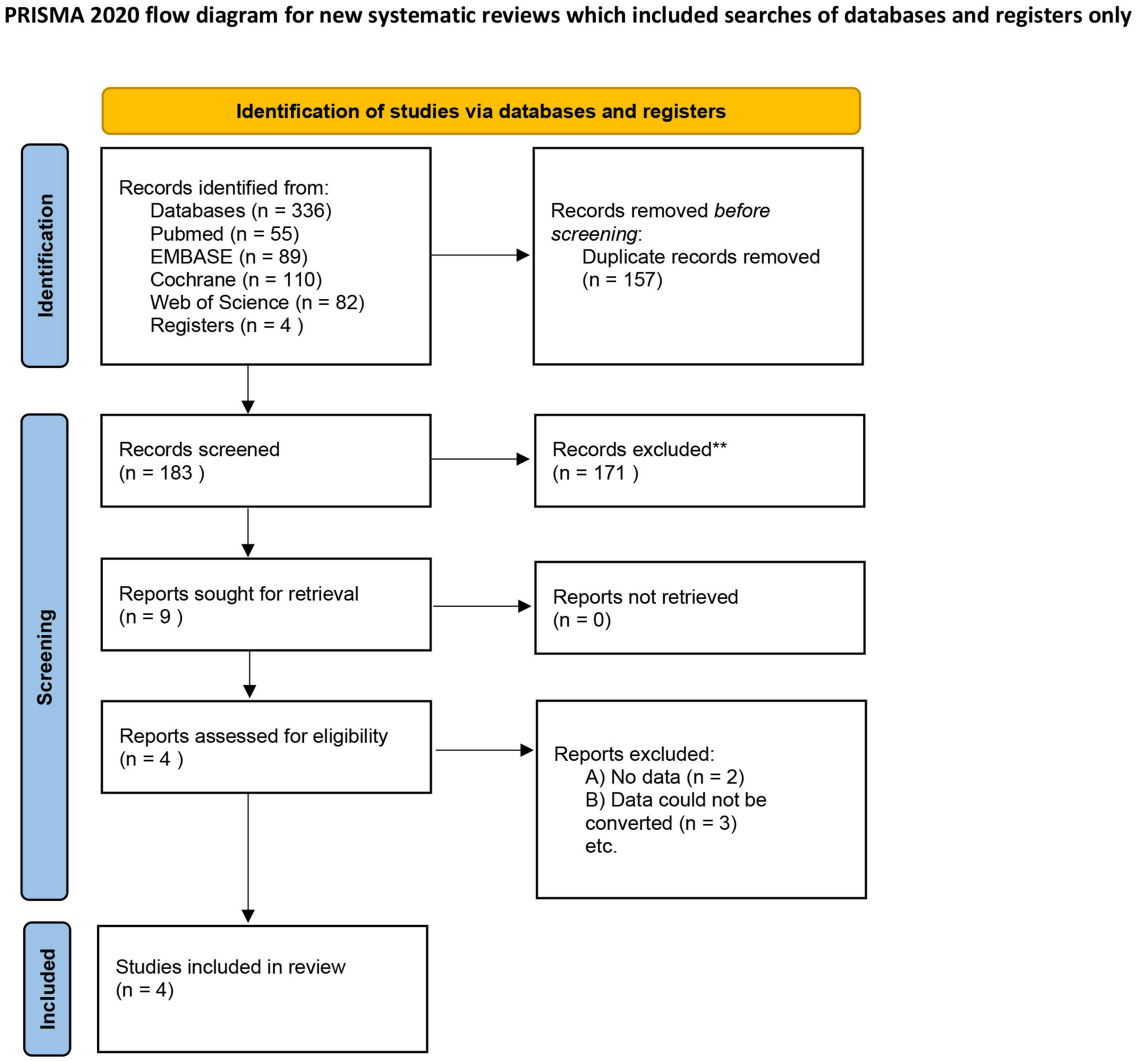
PRISMA 2020 flow diagram of the study selection process. This diagram illustrates the systematic literature search and selection workflow. A total of 340 records were identified from PubMed, Embase, Cochrane CENTRAL, and Web of Science. After removing duplicates (*n* = 157) and screening titles/abstracts, 9 full-text articles were assessed for eligibility. Four RCTs met the final inclusion criteria (PICO: low-dose vs. conventional-dose isotretinoin for acne) and were included in the quantitative meta-analysis.

Database searches identified 340 records. After removing duplicates and screening, four RCTs met inclusion criteria: Lee et al. (24), Kassem et al. (25), Faghihi et al. (28), and El Aziz Ragab et al. (26), comprising 210 randomized patients (202 analyzed) (Figure 1). Table 1 summarizes trial characteristics. All studies evaluated 24-week treatment periods, though follow-up durations varied (6–12 months). Notably, Faghihi et al. (28) enrolled patients with higher baseline severity and used an adjunctive protocol (short courses of oral prednisolone and azithromycin at initiation, then topical clindamycin maintenance), representing potential co-interventions that may contribute to between-study heterogeneity.

**Table 1:** Characteristics of included randomized controlled trials.

Characteristic	Kassem et al. (25)	Faghihi et al. (28)	Lee et al. (24)	El Aziz Ragab et al. (26)
Country	Syria	Iran	Korea	Egypt
Study design	RCT	RCT	RCT	RCT
Sample size^a^	72 (LD: 38; CD: 34)	60 (LD: 30; CD: 30)	40 (LD: 20; CD: 20)	30 (LD: 15; CD: 15)
Population	Moderate acne	Moderate to severe acne	Moderate acne	Moderate to severe acne
Mean age ± SD (years)	LD: 21.8 ± 4.6; CD: 20.1 ± 2.5	LD: 22.9 ± 6.3; CD: 23.1 ± 4.7	LD: 23.6 ± 3.4; CD: 20.8 ± 2.7	LD: 20.0 (17–24); CD: 21.0 (17–24)^c^
Sex (M/F)	LD: 11/27; CD:13/21	LD: 5/25; CD:8/22	LD: 5/12; CD:6/10^b^	LD: 11/4; CD: 11/14
Baseline GAGS score	LD: 24.27 ± 3.20; CD: 25.44 ± 3.79	LD: 54.6 ± 2.9; CD: 58.8 ± 3.1	LD: 25.80 ± 3.55; CD: 25.85 ± 2.76	LD: 29.0 ± 6.6; CD: 28.0 ± 6.5
Intervention (low-dose)	0.25–0.4 mg/kg/day	0.25 mg/kg/day	0.25–0.4 mg/kg/day	20 mg/day
Comparator (conventional-dose)	0.5–1.0 mg/kg/day	0.5 mg/kg/day	0.5–0.7 mg/kg/day	0.5 mg/kg/day
Treatment duration	24 weeks	24 weeks (6 months)	24 weeks	24 weeks (6 months)
Post-treatment follow-up	6 months	6 months	12 months	12 months
Primary outcomes	GAGS score	GAGS score	GAGS score	GAGS score
Secondary outcomes	Adverse events, patient satisfaction	Adverse events, patient satisfaction	Adverse events, patient satisfaction	

Data are presented as mean ± standard deviation or *n* (%) unless otherwise specified. CD, conventional-dose; GAGS, Global Acne Grading System; IQR, interquartile range; LD, low-dose; RCT, randomized controlled trial. Kassem et al. (25) and Lee et al. (24) originally randomized patients to three arms; only the conventional-dose and low-dose arms were included in this meta-analysis to align with inclusion criteria. In Faghihi et al. (28), all participants received adjunctive therapy at initiation: oral prednisolone (0.25 mg/day) for 1 week and oral azithromycin (250 mg/day) for 2 weeks, followed by maintenance with 2% topical clindamycin.

a Sample sizes reflect the number of participants analyzed in the two-arm comparison.

b Gender distribution for Lee et al. (24) represents the per-protocol population (post-dropout).

c Age reported as median (IQR).

### 3.2 Risk of bias assessment

**Figure 2:**
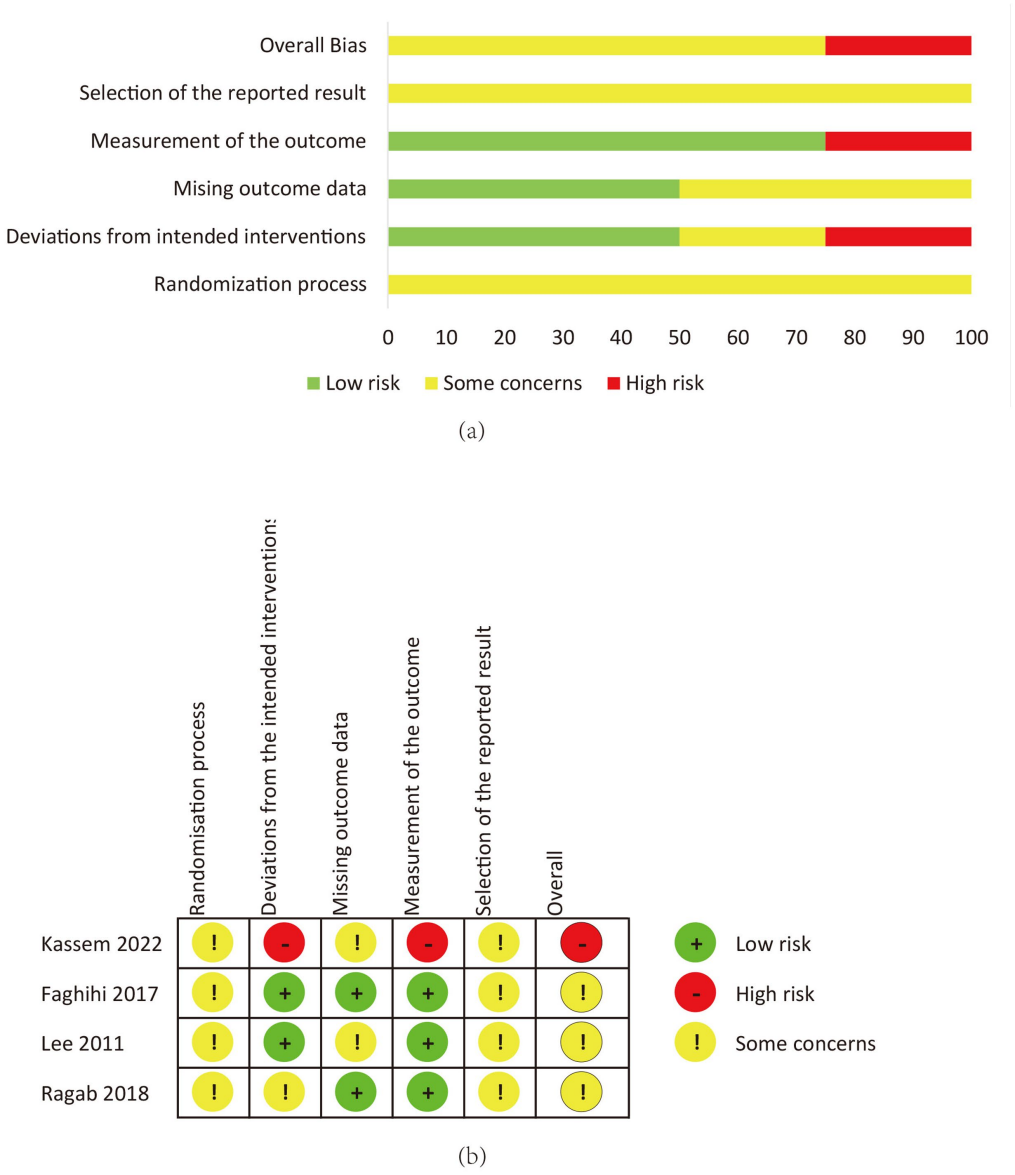
Risk of bias (RoB 2) assessment at the outcome level. **(a)** Domain-level summary graph showing the proportion of studies with “Low risk,” “Some concerns,” or “High risk” of bias across the five RoB 2 domains for the primary outcome (24-week GAGS change). **(b)** “Traffic-light” plot presenting individual risk judgments for each included study. Green circles indicate low risk, yellow indicates some concerns, and red indicates high risk. The overall rating was influenced by concerns regarding measurement bias (lack of blinding in Kassem et al. (25)) and randomization reporting (Faghihi et al. (28)).

Blinded outcome assessment was reported in Lee et al. (24), Faghihi et al. (28), and Kassem et al. (25) did not report blinding; El Aziz Ragab et al. (26) was double-blind but sparingly reported. Overall, three trials had “some concerns” and one had “high risk” (measurement bias). Detailed domain-level judgments are presented in Figure 2.

### 3.3 Treatment dynamics (0–24 weeks)

**Figure 3:**
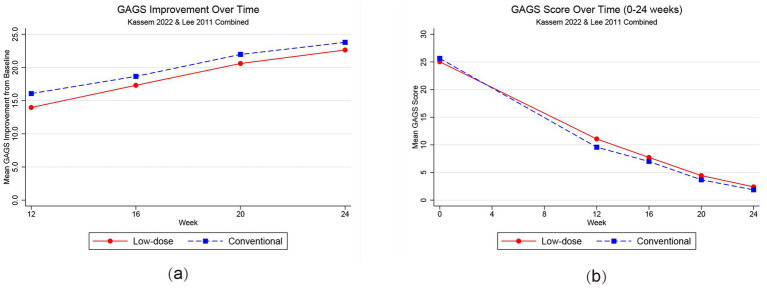
Temporal dynamics of GAGS score improvement over 24 weeks. **(a)** Mean reduction in GAGS scores from baseline at each follow-up visit. **(b)** Absolute GAGS scores over the treatment course. The red solid line represents the low-dose group, and the blue dashed line represents the conventional-dose group. Error bars represent standard deviations. Intermediate time-point between-group comparisons of GAGS improvement (change from baseline) at weeks 12, 16, and 20 are provided in Supplementary Figure S1. These analyses suggest a modest early advantage with conventional dosing, based on two trials reporting intermediate assessments.

Analysis of studies providing multi-timepoint data (Lee et al. (24) and Kassem et al. (25)) revealed differences in treatment kinetics (Figure 3). Two RCTs reported intermediate GAGS assessments during the 24-week treatment course (Lee et al. (24) and Kassem et al. (25)). In pooled random-effects analyses of GAGS improvement (change from baseline), conventional dosing was associated with greater early improvement at week 12 [MD = −2.20, 95% CI (−3.34, −1.07); *p* < 0.001], week 16 [MD = −1.48, 95% CI (−2.60, −0.36); *p* = 0.01], and week 20 [MD = −1.46, 95% CI (−2.80, −0.12); *p* = 0.03] (Supplementary Figure S1). However, this advantage diminished progressively over the course of therapy, with outcomes converging to comparable levels by week 24. These intermediate time-point estimates were based on two trials and should be interpreted as supportive rather than definitive.

### 3.4 Treatment efficacy at 24 weeks

**Figure 4:**
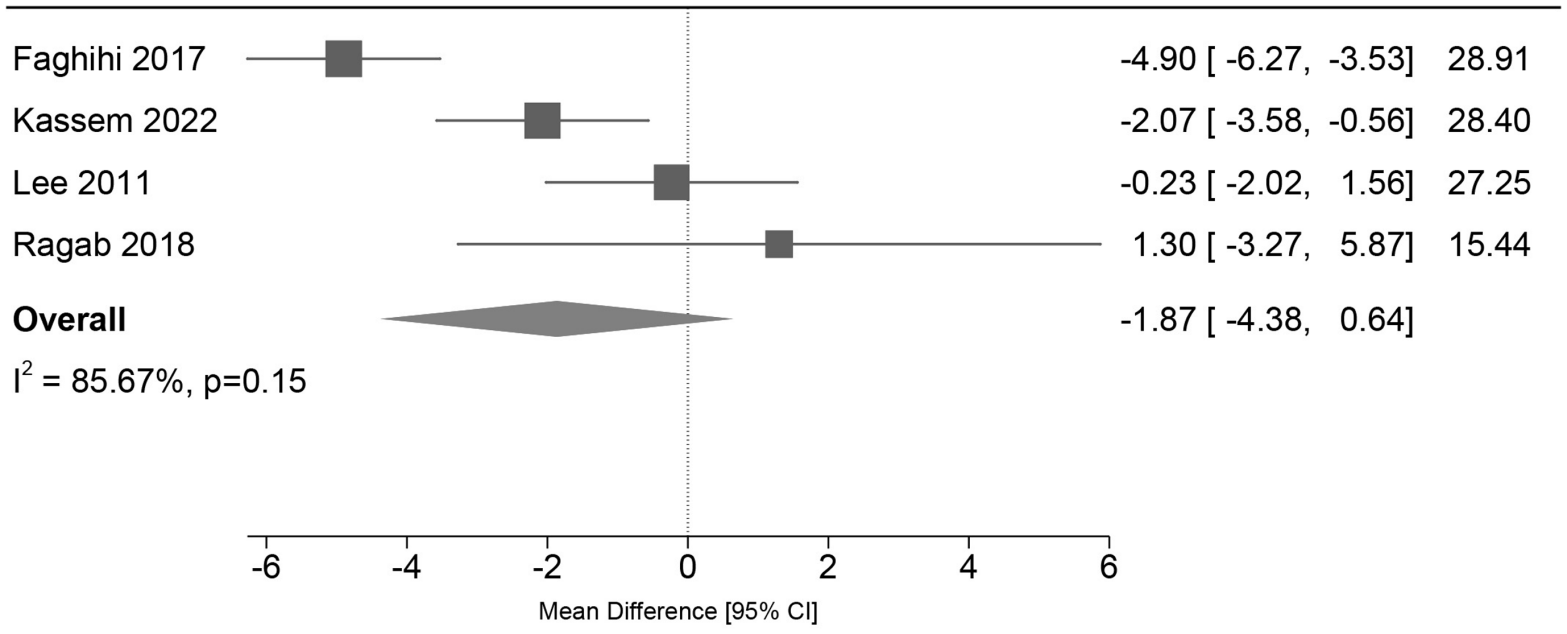
Forest plot of the primary outcome: GAGS score reduction at 24 weeks (All studies). Comparison of MD in GAGS score reduction between low-dose and conventional-dose regimens using a random-effects model. Individual study effect sizes are represented by squares, with the size proportional to the study’s weight. Horizontal lines indicate 95% confidence intervals (CI). The diamond represents the pooled MD. The vertical line at 0 indicates no difference; values to the left favor conventional dosing, and values to the right favor low dosing. High heterogeneity (*I*^2^ = 85.67%) was observed, and the overall difference was not statistically significant (*p =* 0.15).

**Figure 5:**
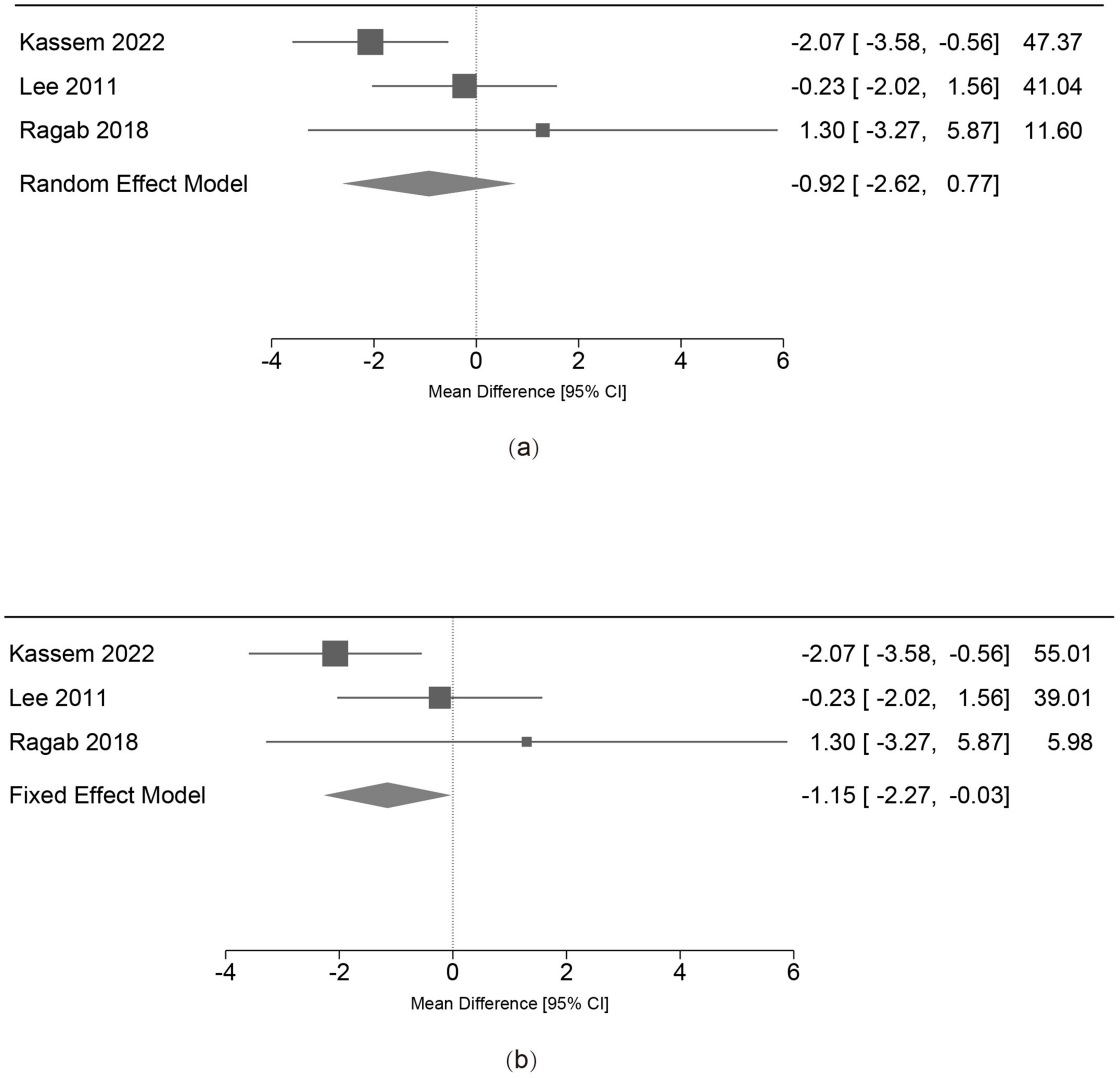
Sensitivity analysis forest plots for 24-week GAGS reduction (Excluding Faghihi et al. (28)). This analysis excludes Faghihi et al. (28) to address heterogeneity caused by baseline severity differences and adjunctive therapies. **(a)** Random-effects model: Heterogeneity decreased significantly (*I*^2^ = 44.97%), and no statistically significant difference was found between dosing regimens (*p =* 0.28). **(b)** Fixed-effect model: Under fixed-effect assumptions, a marginal statistical difference (MD = −1.15, *p* = 0.04) favored the conventional-dose group. However, the absolute difference (~1-2 GAGS points) is small and its clinical importance is uncertain in the absence of a validated MCID for GAGS.

All regimens demonstrated substantial GAGS score improvements. Primary meta-analysis revealed no significant difference in GAGS reduction between groups at 24 weeks [MD = −1.87, 95% CI (−4.38, 0.64), *p =* 0.15], though heterogeneity was high (*I*^2^ = 85.67%) (Figure 4). Leave-one-out analyses indicated that heterogeneity was predominantly attributable to Faghihi et al. (28) excluding any other trial did not materially alter the pooled estimate (Figure 4) (Supplementary Figure S2). This heterogeneity is consistent with clinical differences in baseline severity and co-interventions in that study. In Faghihi et al. (28) mean baseline GAGS was approximately 55–59, well above the very-severe threshold in commonly used GAGS categorizations. In addition, all participants received systemic and topical co-interventions (azithromycin for 2 weeks and prednisolone during the first week, followed by topical clindamycin during follow-up), which may have modified early response and post-treatment course. Upon exclusion of this study, heterogeneity declined to moderate levels (*I*^2^ = 44.97%), and the random-effects model maintained a non-significant difference [MD = −0.92, 95% CI (−2.62, 0.77), *p =* 0.28] (Figure 5a). While a fixed-effect model excluding Faghihi et al. (28) suggested a marginal advantage for conventional dosing [MD = −1.15, 95% CI (−2.27, −0.03), *p =* 0.04] (Figure 5b). The absolute magnitude (approximately 1-2 GAGS points) is small and its clinical importance is uncertain. Moreover, given the width of commonly used GAGS severity categories (e.g., moderate 19–30; severe 31–38; very severe ≥39), a 1-2 point difference is unlikely to translate into a meaningful shift in severity classification for most patients. Finally, varying the assumed correlation coefficient for standard deviation imputation (*r* = 0.3 and *r* = 0.7) did not impact the direction or statistical significance of the primary outcome.

### 3.5 Post-treatment outcomes

Post-treatment outcomes (quantified as GAGS score deterioration) were analyzed in three RCTs reporting scores at follow-up (6–12 months). El Aziz Ragab et al. (26) defined relapse dichotomously and was excluded from the continuous data meta-analysis. In the pooled analysis of all three studies, there was no statistically significant difference in GAGS score deterioration between the low-dose and conventional-dose groups [MD = −0.85, 95% CI (−2.40, 0.70), *p =* 0.28] (Supplementary Figure S3a). However, substantial heterogeneity was observed (*I*^2^ = 55.60%), largely driven by Faghihi et al. (28). In this trial, the conventional-dose group exhibited a greater magnitude of score rebound (+7.9 points) compared to the low-dose group (+6.2 points). To address this, a sensitivity analysis was performed excluding Faghihi et al. (28) to reduce the influence of relevant co-interventions (maintenance topical clindamycin and short-course systemic agents) used in that trial. The remaining analysis, pooling Kassem et al. (25) (6 month follow-up) and Lee et al. (24) (12 month follow-up), demonstrated zero heterogeneity (*I*^2^ = 0.00%) and a negligible difference between dosing regimens [MD = 0.10, 95% CI (−1.67, 1.86), *p =* 0.91] (Supplementary Figure S3b). This suggests that observed post-treatment outcome differences may be sensitive to co-interventions and clinical context rather than dose alone. Because relapse definitions varied across trials (continuous GAGS deterioration vs. retreatment-based or threshold-based relapse), these results should be interpreted as post-treatment changes in severity scores and may not be directly comparable with relapse rates defined by retreatment need.

### 3.6 Adverse events

Mucocutaneous adverse events (AEs) were consistently dose-dependent. In Lee et al. (24), cheilitis occurred in 93.8% of the conventional-dose group versus 64.7% of the low-dose group; xerosis (31.3% vs. 5.9%) and epistaxis (18.8% vs. 0%) were also more frequent with conventional dosing. Where reported, laboratory abnormalities and adverse-event–related discontinuations occurred in conventional-dose arms; estimates are limited by small numbers and selective reporting across trials. In Faghihi et al. (28), nasal dryness (40.0% vs. 20.0%) and hair thinning (23.3% vs. 6.7%) were more common with conventional dosing. All patients in this trial were narratively reported to have dry lips, preventing comparative risk calculation for cheilitis. Kassem et al. (25) reported “any AE” in 100% of conventional-dose patients versus 84% of low-dose patients, with reversible laboratory elevations confined to the conventional arm (Table 2).

**Table 2:** Summary of key adverse events and tolerability outcomes by regimen.

Adverse event	Faghihi et al. (28) LD (*n* = 30)	Faghihi et al. (28) CD (*n* = 30)	Lee et al. (24) LD (*n* = 17)	Lee et al. (24) CD (*n* = 16)	Kassem et al. (25) LD (*n* = 38)	Kassem et al. (25) CD (*n* = 34)	El Aziz Ragab et al. (26)
Any adverse event	—	—	11/17 (64.7%)	15/16 (93.8%)	32/38 (84.2%)	34/34 (100%)	NR
AE-related discontinuation	—	—	0/17 (0%)	2/16 (12.5%)	0/38 (0%)	0/34 (0%)	NR
Cheilitis/dry, chapped lips	Narrative: 100%^†^	Narrative: 100%^†^	11/17 (64.7%)	15/16 (93.8%)	NR	NR	NR
Xerosis (dry skin)	NR	NR	1/17 (5.9%)	5/16 (31.3%)	NR	NR	NR
Nasal symptoms	Dry nose 6/30 (20.0%); Repeated rhinorrhea 0/30 (0%)	Dry nose 12/30 (40.0%); Repeated rhinorrhea 6/30 (20.0%)	Epistaxis 0/17 (0%)	Epistaxis 3/16 (18.8%)	NR	NR	NR
Dry eyes	4/30 (13.3%)	6/30 (20.0%)	NR	NR	NR	NR	NR
Hair thinning/loss	2/30 (6.7%)	7/30 (23.3%)	NR	NR	NR	NR	NR
Musculoskeletal (myalgia/arthralgia)	Myalgia 0/30 (0%); Arthralgia 2/30 (6.7%)	Myalgia 1/30 (3.3%); Arthralgia 1/30 (3.3%)	NR	NR	NR	NR	NR
Laboratory abnormalities	NR	NR	Triglycerides↑ 0/17 (0%); AST/ALT↑ 0/17 (0%)	Triglycerides↑ 1/16 (6.3%); AST/ALT↑ 1/16 (6.3%)	Triglycerides↑ 0/38 (0%); AST/ALT↑ 0/38 (0%)	Triglycerides↑ 2/34 (5.9%); AST/ALT↑ 1/34 (2.9%)	NR

NR, not reported. Nasal events differ in definition (epistaxis vs. dryness/rhinorrhea) and are displayed side-by-side. AE-related discontinuation occurred only in the Lee et al. (24) conventional arm (2/16). Kassem et al. (25) reported “Any AE” as percentages (LD 84% vs. CD 100%); counts were derived for visualization. † Faghihi et al. (28) narratively reported universal dry lips (retinoid cheilitis) but did not provide per-event counts; thus, data were not pooled with other trials.

### 3.7 Patient satisfaction

**Figure 6:**
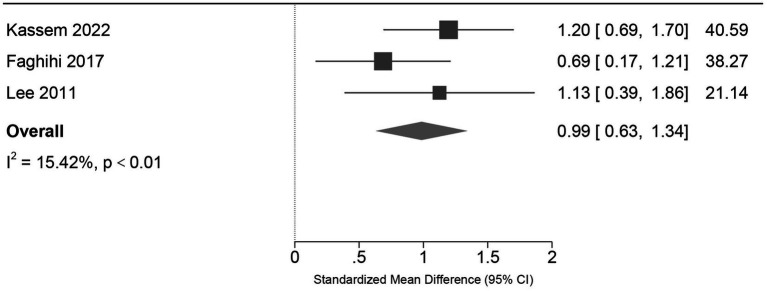
Forest plot of patient satisfaction scores. Comparison of standardized mean differences (SMD) for patient-reported satisfaction at the end of treatment. Since studies used different scales (Likert scale vs. Visual Analog Scale), SMD was used to harmonize the data. Positive values favor the low-dose regimen. The analysis demonstrates a large, statistically significant preference for low-dose therapy [SMD = 0.99, 95% CI (0.63, 1.34), *p* < 0.001] with low heterogeneity (*I*^2^ = 15.42%), likely reflecting the superior tolerability profile.

Patient satisfaction was reported in three studies using different scales (4 point Likert vs. 0–5 VAS). After harmonizing directionality, the pooled analysis demonstrated a large, statistically significant preference for the low-dose regimen [SMD = 0.99, 95% CI (0.63, 1.34), *p* < 0.001], with low heterogeneity (*I*^2^ = 15.4%) (Figure 6).

## 4 Discussion

This systematic review and meta-analysis suggests that, in the available RCTs, low-dose isotretinoin achieves 24-week efficacy comparable to conventional dosing while offering a more favorable tolerability profile—particularly in moderate acne and when co-interventions are minimized. Conventional dosing did show a modest early advantage in GAGS improvement at weeks 12–20, but the absolute differences were small (approximately 1-2 GAGS points) and had largely disappeared by week 24. In the absence of a validated MCID for GAGS, the clinical importance of these transient differences remains uncertain, and the most directly applicable evidence for dose de-escalation without loss of 24-week efficacy comes from trials restricted to moderate acne and minimizing concomitant therapies; whether these findings extend to substantially more severe disease remains to be determined.

Post-treatment outcomes were broadly comparable between dosing regimens. After exclusion of the confounded trial by Faghihi et al. (28) (adjunctive systemic antibiotics, corticosteroids and maintenance topical clindamycin), pooled data from Kassem et al. (25) (6 month follow-up) and Lee et al. (24) (12 month follow-up) showed no meaningful difference in post-treatment GAGS deterioration between low-dose and conventional-dose isotretinoin. As both trials enrolled patients with moderate acne, these findings suggest that, in moderate acne, long-term relapse severity does not differ significantly between low-dose and conventional-dose regimens when co-interventions are minimized; however, certainty remains limited by small samples and study-level heterogeneity.

The large and consistent advantage in patient satisfaction with low-dose therapy (SMD 0.99) is clinically important. Adherence to isotretinoin is frequently limited by mucocutaneous adverse effects (29), and our analysis confirms a clearly better tolerability profile with low-dose regimens. By reducing this burden, low-dose strategies may support better treatment acceptability and adherence; whether this translates into improved long-term outcomes warrants confirmation in larger, pragmatic trials.

A key observation from the included trials is that low-dose isotretinoin achieved comparable efficacy with cumulative doses of approximately 60 mg/kg—well below the traditional 120–150 mg/kg target. This accords with Tan et al.’s conclusion that the conventional cumulative-dose range is not strongly evidence-based (30). Practice-based data from Rademaker similarly indicate that even patients treated with very low cumulative doses (e.g., 25–50 mg/kg) do not relapse more often than those receiving higher exposures, provided treatment is continued until complete clearance and for at least two additional months, and that relapse risk appears to relate more closely to the duration of sebaceous gland suppression than to attainment of an arbitrary cumulative-dose threshold (12, 31). Taken together, the available RCT evidence—conducted predominantly in moderate acne and in contexts minimizing co-interventions— is most consistent with an individualized dosing strategy, in which daily dose and treatment duration are tailored to baseline severity, tolerability, and patient goals, with emphasis on achieving clinical clearance and adequate suppression duration. However, the small number and heterogeneity of trials—and low certainty of evidence for efficacy—preclude definitive dose–response conclusions or practice-changing recommendations regarding long-term relapse prevention.

We applied the GRADE framework to assess the certainty of evidence (Table 3). Certainty for efficacy was rated low due to heterogeneity and sensitivity to study context; certainty for safety and satisfaction was moderate. This distinction is clinically relevant: while the precise magnitude of efficacy equivalence requires further homogeneous data, the tolerability advantage of low-dose regimens is supported by robust evidence, strengthening the rationale for its use as a patient-centered alternative.

**Table 3:** Summary of findings (GRADE assessment) low-dose vs. conventional-dose isotretinoin for moderate-to-severe acne vulgaris.

Outcomes	Number of participants (studies)	Effect estimate (95% CI)	Certainty of the evidence (GRADE)	Summary
Clinical efficacy (change in GAGS at end of treatment)	202 (4 RCTs)	MD	Low^a^^,^^b^	Low-dose regimens likely result in little to no difference in acne severity reduction compared to conventional dosing.
		−1.87 (−4.38 to 0.64)		
Patient satisfaction (end of treatment)	149 (3 RCTs)	SMD	Moderate^c^	Low-dose regimens are associated with significantly higher patient satisfaction scores.
		0.99 (0.63 to 1.34)		
Adverse events (mucocutaneous events like cheilitis, xerosis)	202 (4 RCTs)	Narrative synthesis (see comment)	Moderate^c^	There is moderate certainty that low-dose regimens reduce the frequency and severity of mucocutaneous events.
Relapse (change in GAGS between end of treatment and follow-up)	168 (3 RCTs)	MD	Low^a^^,^^d^	The difference in relapse rates remains uncertain due to wide CIs and confounding factors in primary studies.
		−0.85 (−2.40 to 0.70)		

a Downgraded one level for risk of bias: concerns regarding randomization concealment [e.g., Faghihi et al. (28)] or lack of blinding in outcome assessment [e.g., Kassem et al. (25)].

b Downgraded one level for inconsistency: substantial heterogeneity (*I*^2^ = 85.67%) was observed in the primary analysis which could not be fully explained by sensitivity analysis.

c Downgraded one level for risk of bias: subjective outcomes (satisfaction, side effect tolerability) in open-label designs are susceptible to performance and detection bias.

d Downgraded one level for imprecision: the 95% CI crosses the line of no effect and includes possibilities of both benefit and harm.

Several limitations of this review should be considered when interpreting these findings. First, risk of bias was not negligible (three trials had some concerns and one was at high risk), and certainty of evidence was low for efficacy and moderate for safety and satisfaction (GRADE). Second, the evidence base was small, and some outcomes were sensitive to single studies, limiting precision and generalizability. Third, clinically important co-interventions varied across trials—most notably systemic antibiotics/corticosteroids and topical maintenance therapy in Faghihi et al. (28)—complicating attribution of effects to isotretinoin dose alone. Meta-regression was considered to explore baseline severity as a source of heterogeneity; however, with only four included trials, such analyses would be underpowered and potentially unstable. Future RCTs should standardize adjunctive therapies, adopt clinically comparable relapse definitions, and enroll patients across a broader range of acne severity to clarify whether these findings extend beyond moderate disease.

## 5 Conclusion

Low-dose isotretinoin provides comparable 24-week efficacy to conventional dosing with better tolerability and higher patient satisfaction in randomized trials conducted predominantly in moderate acne and in contexts minimizing co-interventions. Post-treatment GAGS deterioration over 6–12 months did not differ significantly between regimens in trials minimizing co-interventions, although certainty is limited. Given limited and heterogeneous evidence, dosing should be individualized based on baseline severity, achievement of clinical clearance, suppression duration, and patient preferences. Conventional dosing may remain appropriate for patients with higher baseline severity or a need for faster early response, whereas low-dose regimens may be preferred when tolerability and adherence are paramount. Larger, more homogeneous RCTs with longer follow-up are needed to clarify long-term relapse and dose–response relationships.

## Data Availability

The original contributions presented in the study are included in the article/Supplementary material, further inquiries can be directed to the corresponding authors.
