# Supplementary material for: Individualizing isotretinoin dosing in acne: comparable 24-week efficacy and better tolerability at lower daily doses
Source: Front Med (Lausanne). 2026 Mar 9;13:1771320. doi: 10.3389/fmed.2026.1771320 (PMC13006244; doi:10.3389/fmed.2026.1771320)
Supplement: Supplementary file 1 [file Image_1.pdf]

Figure S1. Intermediate time-point meta-analyses of early treatment kinetics (GAGS improvement): low-dose vs conventional-dose isotretinoin.

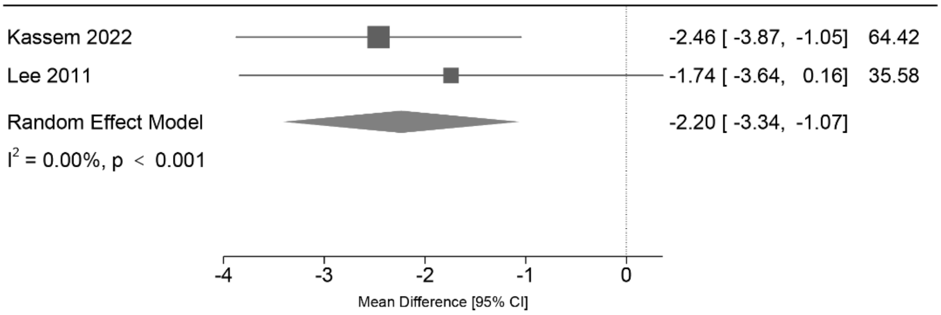

(a)

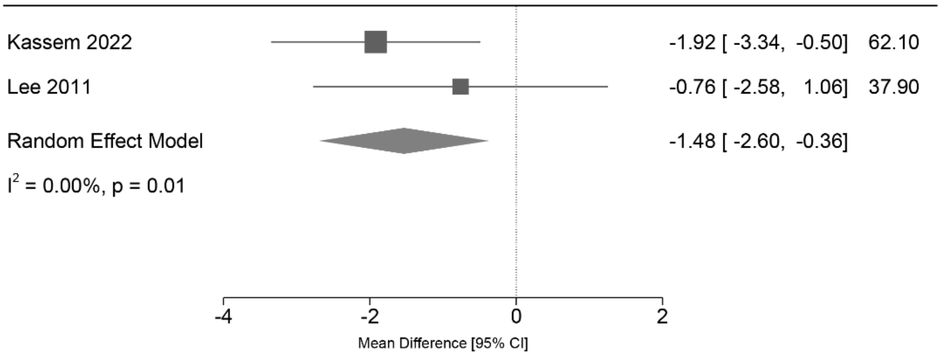

(b)

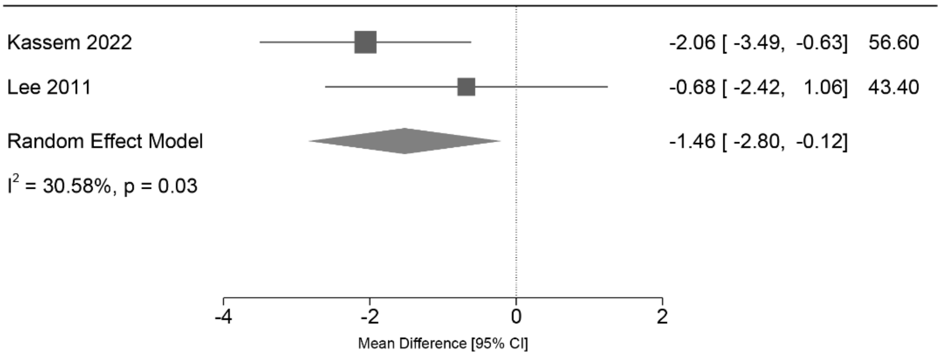

(c)

Forest plots summarize between-group differences in GAGS improvement (change from baseline) at week 12 (S3A), week 16 (S3B), and week 20 (S3C) during the 24-week treatment course. The pooled effect is the mean difference (MD) calculated as low-dose minus

conventional-dose; thus, MD < 0 favors conventional dosing (greater early improvement). Intermediate assessments were available from Lee et al. (4-weekly visits) and Kassem et al. (GAGS assessed at each visit).
