# Supplementary figures and images for: Individualizing isotretinoin dosing in acne: comparable 24-week efficacy and better tolerability at lower daily doses
Source: Front Med (Lausanne). 2026 Mar 9;13:1771320. doi: 10.3389/fmed.2026.1771320 (PMC13006244; doi:10.3389/fmed.2026.1771320)

**Figure S2. Sensitivity analysis forest plots for 24-week GAGS reduction (leave-one-out analysis).**

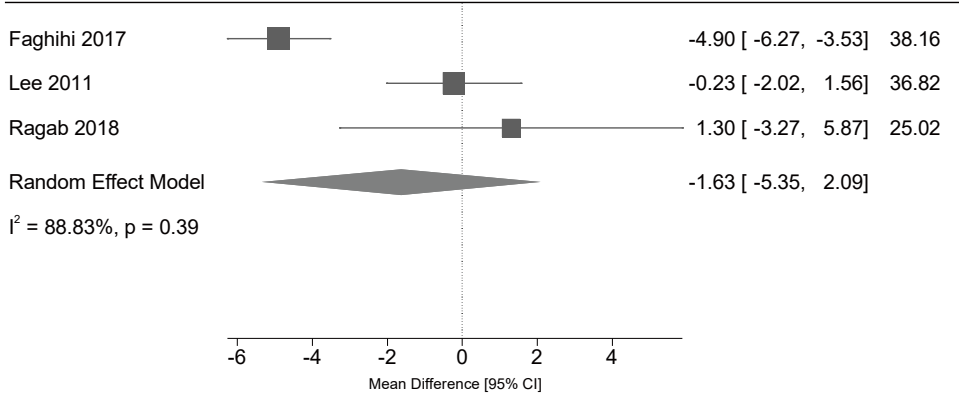

(a)

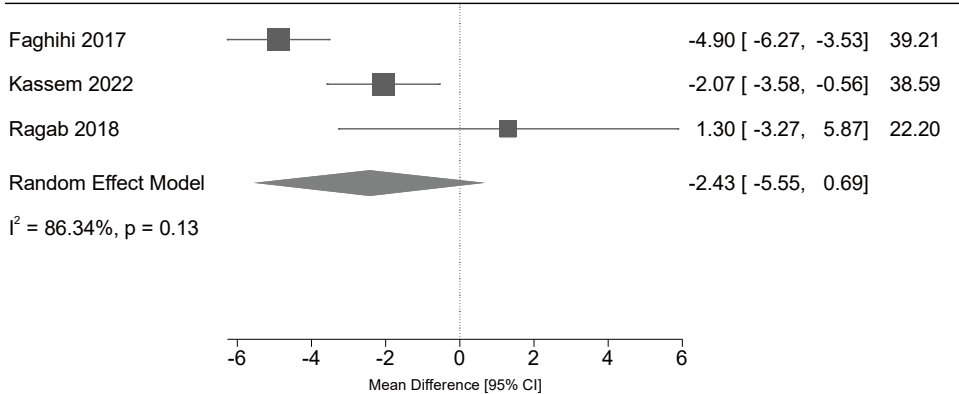

(b)

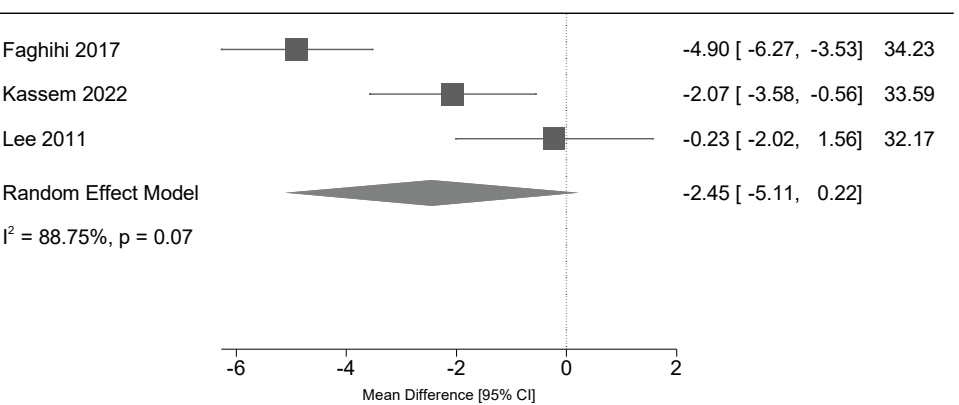

(c)

Supplement: Supplementary file 2 [file Image_2.pdf]
