# Supplementary material for: Individualizing isotretinoin dosing in acne: comparable 24-week efficacy and better tolerability at lower daily doses
Source: Front Med (Lausanne). 2026 Mar 9;13:1771320. doi: 10.3389/fmed.2026.1771320 (PMC13006244; doi:10.3389/fmed.2026.1771320)
Supplement: Supplementary file 3 [file Image_3.pdf]

Figure S3. Sensitivity analysis of relapse severity (GAGS score deterioration from end-of-treatment to follow-up).

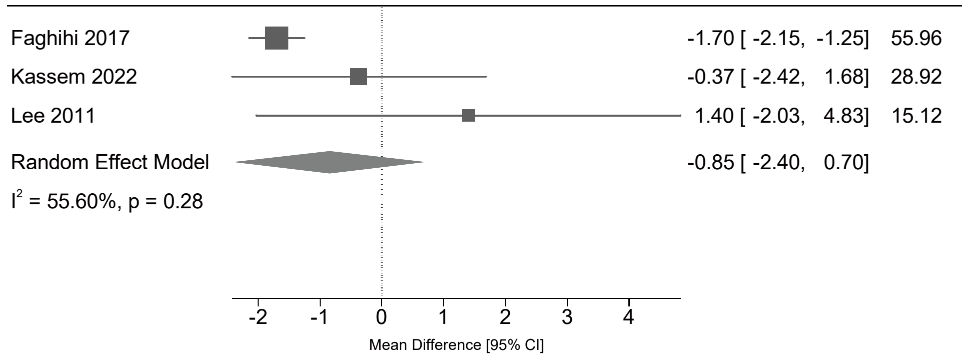

(a)

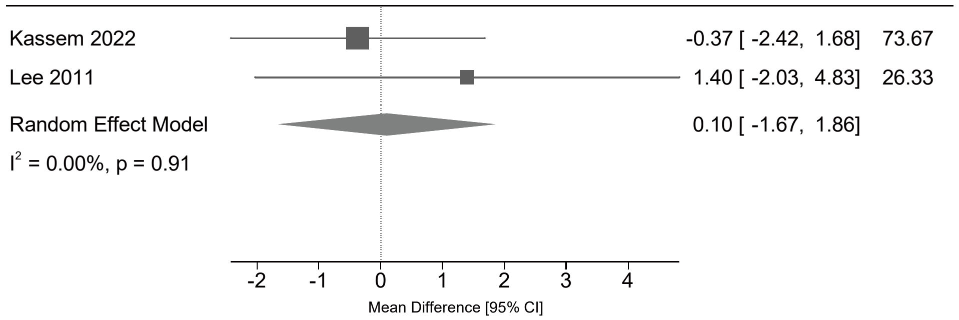

(b)

(a) All Studies Combined (n=3): Forest plot including all RCTs reporting post-treatment GAGS scores (Faghihi et al., Kassem et al., Lee et al.). Note the substantial heterogeneity ($I^2 = 55.60\%$) and the significant weight of Faghihi et al., which shifts the result favoring the low-dose group.

(b) Sensitivity Analysis (Moderate Acne / Excluding Confounders): Forest plot excluding Faghihi et al. (due to baseline severity differences and the confounding use of adjunctive antibiotics/corticosteroids). This analysis pools Kassem et al. (6-month follow-up) and Lee et al. (12-month follow-up). Notably, heterogeneity dropped to $I^2 = 0.00\%$, and the difference between regimens became non-significant (P = 0.91), indicating that the apparent benefit observed in (A) was driven by the excluded study.
