# Supplementary material for: Individualizing isotretinoin dosing in acne: comparable 24-week efficacy and better tolerability at lower daily doses
Source: Front Med (Lausanne). 2026 Mar 9;13:1771320. doi: 10.3389/fmed.2026.1771320 (PMC13006244; doi:10.3389/fmed.2026.1771320)
Supplement: Supplementary file 4 [file Supplementary_file_1.docx]

**Supplementary Appendix 1. Search Strategy**

**Search Date:** May 17, 2025 **Databases:** PubMed, Embase, Cochrane Library, Web of Science, and ClinicalTrials.gov.

**1. PubMed**

**Results:** 55 **Search Query:**  ((("Acne Vulgaris"[Mesh]) OR (Acne[Title/Abstract])) AND (randomized controlled trial[Publication Type] OR randomized[Title/Abstract] OR placebo[Title/Abstract])) AND (("Isotretinoin"[Mesh]) OR (13-cis-Retinoic Acid[Title/Abstract] OR 13 cis Retinoic Acid[Title/Abstract] OR Roaccutane[Title/Abstract] OR Accutane[Title/Abstract] OR Isotretinoin Zinc Salt, 13-cis-Isomer[Title/Abstract] OR Isotretinoin Zinc Salt, 13 cis Isomer[Title/Abstract] OR Ro 4-3780[Title/Abstract] OR Ro 43780[Title/Abstract] OR Ro 4 3780[Title/Abstract])) AND (dose*[Title/Abstract] OR dosing[Title/Abstract] OR dosage[Title/Abstract] OR regimen*[Title/Abstract])

**2. Embase**

**Results:** 89 **Search Query:** (('acne'/exp OR acne) AND vulgaris OR 'acne':ab,ti) AND ('isotretinoin' OR '13-cis-retinoic acid':ab,ti OR '13 cis retinoic acid':ab,ti OR 'roaccutane':ab,ti OR 'accutane':ab,ti OR 'isotretinoin zinc salt, 13-cis-isomer':ab,ti OR 'isotretinoin zinc salt, 13 cis isomer':ab,ti OR 'ro 4-3780':ab,ti OR 'ro 43780':ab,ti OR 'ro 4 3780':ab,ti) AND ('dose*':ab,ti OR 'dosing':ab,ti OR 'dosage':ab,ti OR 'regimen*':ab,ti) AND ('randomized controlled trial':ab,ti OR 'randomized':ab,ti OR 'placebo':ab,ti OR 'rct':ab,ti)

**3. Cochrane Library**

**Results:** 110 **Search Query:** #1 (Acne Vulgaris) OR (acne):ab,ti,kw #2 (Isotretinoin) OR ((13 cis Retinoic Acid):ab,ti,kw OR (Roaccutane):ab,ti,kw OR (Accutane):ab,ti,kw OR (Isotretinoin Zinc Salt, 13 cis Isomer):ab,ti,kw OR (Ro 43780):ab,ti,kw OR (Ro 4 3780):ab,ti,kw) #3 (dose*):ab,ti,kw OR (dosing):ab,ti,kw OR (dosage):ab,ti,kw OR (regimen*):ab,ti,kw #4 (randomized controlled trial):ab,ti,kw OR (randomized):ab,ti,kw OR (placebo):ab,ti,kw OR (RCT):ab,ti,kw **Final Search:** #1 AND #2 AND #3 AND #4

**4. Web of Science**

**Results:** 82 **Search Query:** TS=(Acne Vulgaris OR acne) AND TS=(Isotretinoin OR 13-cis-Retinoic Acid OR 13 cis Retinoic Acid OR Roaccutane OR Accutane OR Isotretinoin Zinc Salt, 13-cis-Isomer OR Isotretinoin Zinc Salt, 13 cis Isomer OR Ro 4-3780 OR Ro 43780 OR Ro 4 3780) AND TS=(dose* OR dosing OR dosage OR regimen*) AND TS=(randomized controlled trial OR randomized OR placebo OR RCT)

**5. ClinicalTrials.gov**

**Results:** 4 **Search Parameters:**

- **Condition or disease:** Acne
- **Intervention/treatment:** Isotretinoin
- **Study Type:** Interventional
- **Status:** With results
